# The Influence of Different Occlusal Loading on Six Restorative Materials for Restoration of Abfraction Lesions—Finite Element Analysis

**DOI:** 10.1055/s-0041-1741376

**Published:** 2022-03-13

**Authors:** Selma Jakupović, Adnan Šehić, Fuad Julardžija, Alma Gavranović-Glamoč, Amela Sofić, Anita Bajsman, Lejla Kazazić

**Affiliations:** 1 Department of Restorative Dentistry and Endodontics, School of Dentistry, University of Sarajevo, Sarajevo, Bosnia and Herzegovina; 2 Department of Radiology Technologies, Faculty of Health Studies, University of Sarajevo, Sarajevo, Bosnia and Herzegovina; 3 Department of Prosthodontics, School of Dentistry, University of Sarajevo, Sarajevo, Bosnia and Herzegovina; 4 Department of Dental Radiology, School of Dentistry, University of Sarajevo, Sarajevo, Bosnia and Herzegovina; 5 Department of Dental Morphology with Dental Anthropology and Forensics, School of Dentistry, University of Sarajevo, Sarajevo, Bosnia and Herzegovina

**Keywords:** occlusal loading, restoration, abfraction lesions, finite element, FEM analysis

## Abstract

**Objectives** The aim of the study was to analyze the occurrence of stress on teeth with abfraction lesions restored with six different restorative materials, and by introducing the tensile strength parameters to calculate the safety factor of the material under the load (ratio between the strength of the material and the maximum stress).

**Materials and Methods** Three-dimensional models of the mandibular premolar are created from a microcomputed tomography images. An abfraction lesion is modeled on the tooth. The stress of the dental tissues and six restorative materials under functional and nonfunctional occlusal loading of 200 (N) are analyzed by finite element method.

**Statistical Analysis** CTAn program 1.10 and ANSYS Workbench (version 14.0) were used for analysis. Results are presented in von Mises stress.

**Results** Oblique loads caused ≈ four times higher stress in restorative materials than the axial ones. It is noticeable that high values of von Mises stress are measured at the bottom of the sharp lesion, even up to 240 MPa, that are significantly reduced after the restoration. The highest stresses at the restorative material are present at the lower (gingival) margin of the restoration. The highest stresses under both types of loads are measured in nanohybrid composite (Tetric EvoCeram, Ivoclar Vivadent). The lowest values of the stress are measured in the flowable composite (Tetric Flow, Ivoclar Vivadent), but at the same time, the highest value of the stress is measured in the surrounding dental tissues on the tooth restored with the flowable composite. The microhybrid composite (Herculite XR, Kerr), with the highest safety factor, is the material that best withstands the stresses it is exposed to. The obtained safety factor did not exceed the critical limit, except for the glass ionomer cement, with the safety factor lower than 1.

**Conclusion** The type of tooth loading has the greatest influence on the intensity of stress. The value of the obtained stresses in the restorative material and dental tissues differ due to the different mechanical properties of the materials. Restoration of noncarious lesions significantly reduces extremely high stress values at their bottom.

## Introduction

Teeth are made of several types of tissues (enamel, dentin, cementum, and pulp) with different mechanical properties, and the analysis of stress within these structures is a very complex process. 1 The distribution of the stress through a certain structure depends on the shape and mechanical properties of the material, as well as the type of the load (angle, duration and intensity of force, contact surface etc. 2 3 ).

The fracture of hard dental tissues and restorative materials is directly related to the intensity of stress in a certain period of time. 4 A special clinical entity, abfraction, is closely related to the action of occlusal forces and the stress of dental tissues. Abfraction lesion is a type of noncarious cervical lesion (NCCL) which represents a microstructural loss of dental tissue caused by the action of occlusal biomechanical forces in the area of the highest stress concentration—cervical region. The author Grippo (1991) referred to such a loss of the hard dental tissue in the cervical region as abfraction to differentiate it from the lesions caused by erosion and abrasion. 5 Abfraction lesions have a typical form of wedge-shaped tissue defect, with sharp inner and outer edges. The incidence of abfraction lesions increases with the age of the patient, which also refers to the component of tissue fatigue over a longer period. 1 6 They can occur on any tooth, but they are frequently found on the mandibular first premolar. 6 7 Specific morphology of this tooth contributes to such a diagnosis. 8

Restorations of cervical lesions can be challenging due to their location (region) because of the specific structural characteristics of the cementoenamel junction (CEJ).

Restoration of abfraction lesion strengthens the tooth structure and protects the enamel from further fragmenting and erosion, reduces dental sensitivity, and improves the dental esthetics which improves the shape and function of the tooth. 9

The development of adhesive restorative materials enabled minimally invasive treatments in the therapy of cervical lesions. In this case, the retention of cervical restorations only depends on adhesion of the restorative material with enamel and dentin, which is in cervical lesions usually sclerotic. Adhesion strength as well as the formation of the hybrid layer is poor on sclerotic dentin. 10

The most used materials for the restoration of cervical lesions are composite materials, compomers and glass ionomer cements (GICs) (conventional or resin—modified). Although the tooth restoration with NCCLs is a long-term problem in dentistry, the causes of their limited retention rate are not completely understood. 11 Follow-up studies performed *in vivo* have showed that the cervical fillings have shorter retention rate compared with other types of fillings. 12 Retention rate of cervical restorations depends on the activity of masticatory forces of different intensity during the functional and parafunctional activities. 13

The aim of this study is to analyze the distribution of stress on teeth with an abfraction lesion during the activity of axial and paraxial forces being restored with six different restorative materials using finite element method (FEM) analysis. Safety factor of the restorative materials, which represents ratio between the strength of the material and the maximum stress, will be examined as well.

## Materials and Methods

**Fig. 1:**
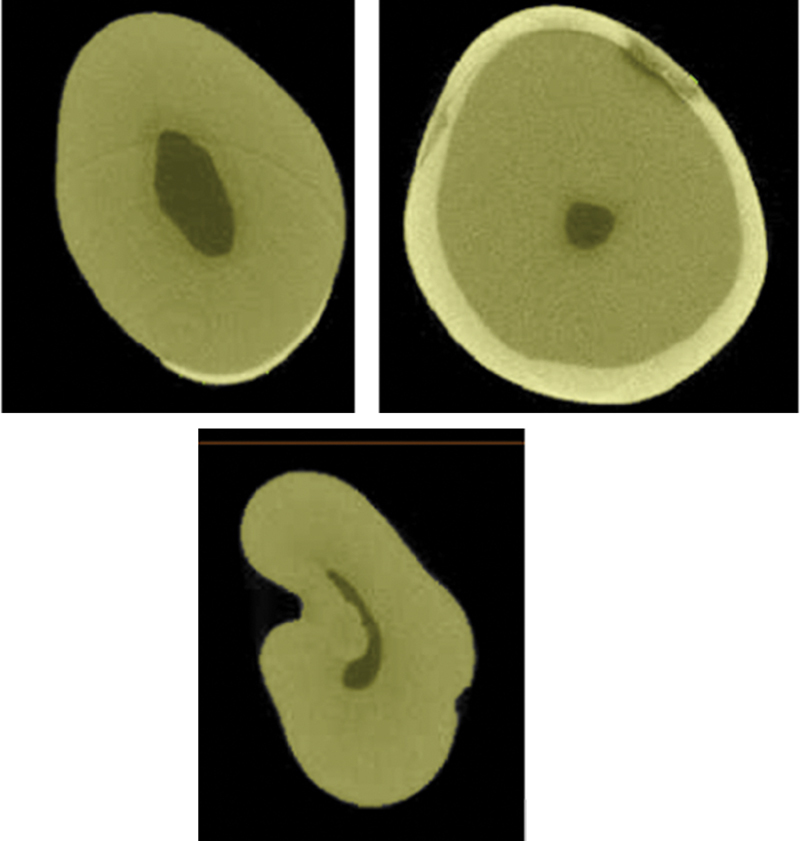
Microcomputed tomography images of the mandibular first premolar.

The mandibular first premolar was selected for the analysis, since the prevalence of NCCLs on this tooth is very high. 6 7 The mandibular first premolar extracted from the orthodontic reasons was scanned on 1076 SkyScan (Kontich, Belgium). The obtained images are reconstructed, using the NRecon program (SkyScan) and analyzed with CTAn program (SkyScan) ( Fig. 1 ).

Data were analyzed in the computer software:

- Computed tomography image processing: Sante DICOM Viewer, MicroDicom, Matlab, µCT software
- CAD model generating: Matlab, CreoParametic 1.0
- FEM analysis: AnsysWorkbench 14.0.

**Fig. 2:**
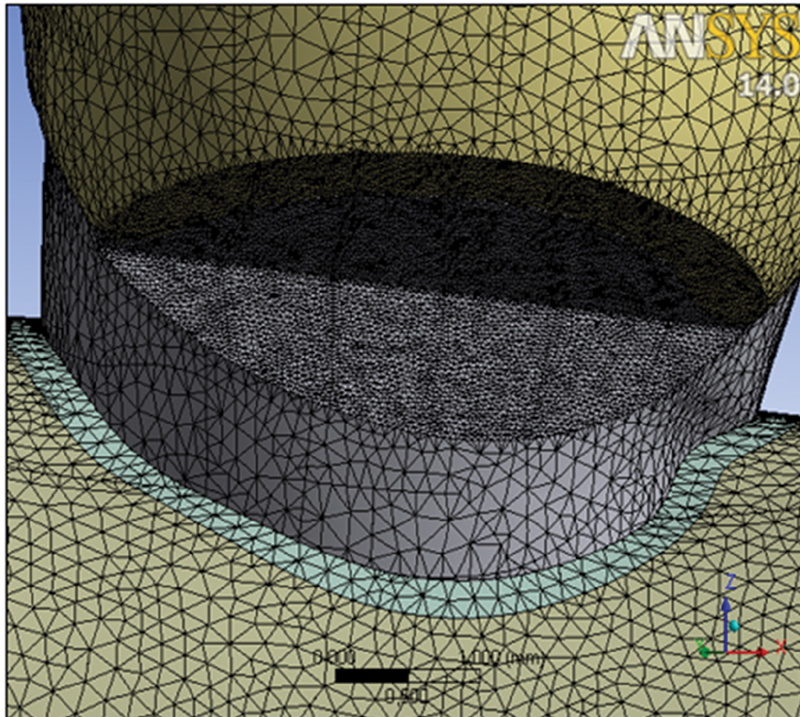
The finite elements mesh.

**Fig. 3:**
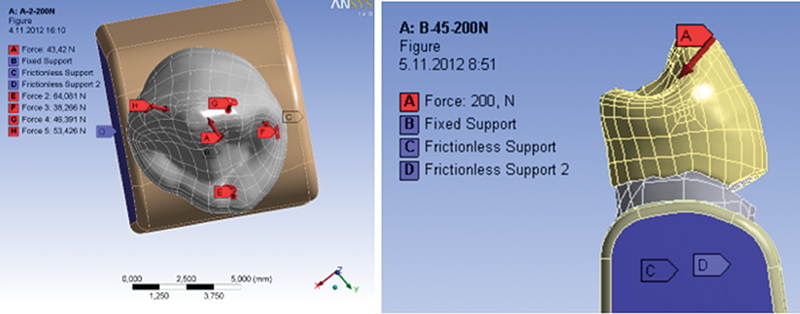
Image of two types of tooth loads.

Using these tools, all dental tissues were reconstructed, and alveolar bone and the abfraction lesion were additionally modeled. The mesh of finite elements has been created, and the tooth model was divided into a large but final number of smaller structural elements( Fig. 2 ). The stress of the simulated axial and oblique load was 200 N 4 8 9 14 ( Fig. 3 ). The model was fixed, so the movement is possible under the load by 300 µm, which is the average thickness of the periodontal membrane. 12

The function of six following restorative materials under load was tested: nanohybrid composite (Tetric EvoCeram, Ivoclar Vivadent), Hybrid composite (Z 100, 3M Espe), Compomer (Dyract, Dentsply Caulk), GIC (Ketac Molar Easy Mix, 3M Espe), Mycrohybrid composite (Herculite XR, Kerr), and flowable composite (Tetric Flow, Ivoclar Vivadent). The adhesive layer of 0.1 mm was modeled as well. The characteristics of the tooth tissues and testing materials are shown in Table 1 .

**Table 1:** Characteristics of materials

Material	Young's modulus of elasticity (MPa)	Poisson's ratio	Literature source
Enamel	84,000	0.30	15
Dentin	18,600	0.31	15
Periodontal ligament	50	0.49	16
Alveolar bone	13,700	0.30	17
Hybrid composite (Z 100, 3M Espe)	15,200	0.28	13
Mycrohybrid composite (Herculite XR, Kerr)	9,500	0.28	18
Nanohybrid composite (Tetric EvoCeram, Ivoclar Vivadent)	17,000	0.28	19
Flowable composite (Tetric flow, Ivoclar Vivadent)	5,300	0.28	13
Compomer (Dyract, Dentsply Caulk)	10,700	0.28	13
Glass ionomer cement (Ketac Molar Easy Mix, 3M Espe)	10,800	0.30	20
Adhesive	1,200	0.25	21

The values of the measured stress are shown by von Mises criteria which are a formula for combining the three principal stresses into an equivalent stress. 22 The equivalent stress is then compared with the ultimate stress of the material to judge the failure condition of the material. The ultimate stress of the material is the maximum stress the material can be loaded by tension, without any breaking. The value of the ultimate stress of the dental tissues and restorative materials are shown in Table 2 .

**Table 2:** Values of the ultimate stress of the enamel, dentin, and restorative materials

Dental tissue and restorative material	Ultimate stress *F*_tu_ (MPa)	Literature source
Enamel	76 MPa, 80 MPa	23
Dentin	103 MPa, 105 MPa	24
Microhybrid composite (Herculite XR, Kerr)	160	25
Hybrid composite (Z 100, 3M Espe)	145	26
Nanohybrid composite (Tetric EvoCeram, Ivoclar Vivadent)	120	26
Flowable composite (Tetric flow, Ivoclar Vivadent)	102	27
Compomer (Dyract, Dentsply Caulk)	93	28
Glass ionomer cement (Ketac Molar Easy Mix, 3M Espe)	45	29

Safety factor was included in the research for the tested restorative materials, and it represents the ratio of strength and stress of the material. To be durable, materials can be loaded only in the elastic area, allowable stresses in the material must not exceed the elastic limit. Materials should not be exposed to their elastic limit but significantly lower. Factor U represents the safety factor, which is calculated as follows:



where ϑ is the safety factor, *F*_tu_ is the ultimate tensile stress, and Ó _allow_ is the allowable stress.

Higher values of the safety factor mean there is less possibility of fracture, while the lower limit is considered to be of a value 1. According to the formula, the safety factor is calculated for all the tested materials.

## Results

All the research results are presented in figures (images) with numeric values of equivalent stress (on the left side of the image).

**Fig. 4:**
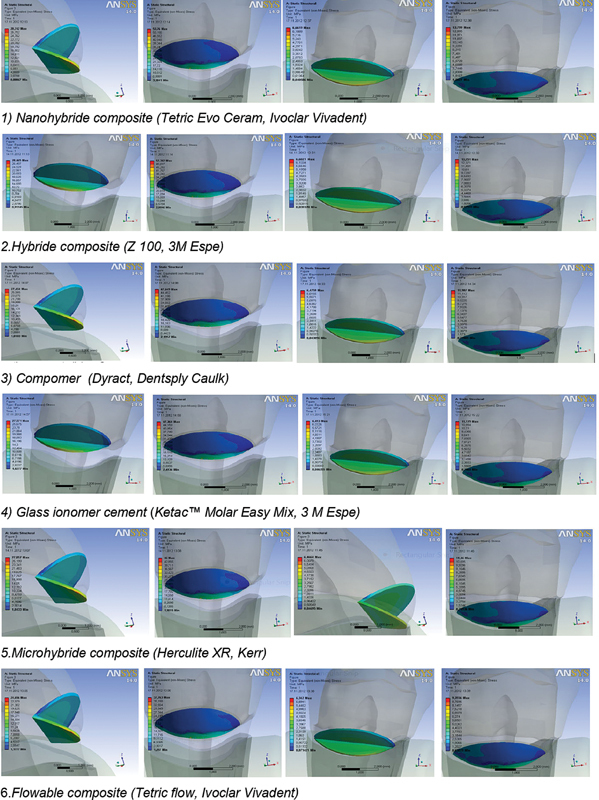
Values of von Mises stress in restorative materials and adhesive system under the axial and oblique load.

Oblique loads cause ≈ four times higher stresses in restorative materials than the axial ones. The highest stresses in the restorative materials and adhesive system are noticed on the lower (gingival) margin of the filling, while the stresses on the upper (occlusal) margin were significantly lower ( Fig. 4 ). The lowest values of the stress were measured in the flowable composite under the axial load of 9.393 MPa and oblique one of 37.763 MPa. The highest values of the stress under both types of the loads were measured in the nanohybrid composite Tetric EvoCeram, Ivoclar Vivadent, and their values were 13.799 and 53.76 MPa, while the values in the adhesive system were 6.661 and 28.742 MPa. Highest values of von Mises stress (up to 240 MPa) are measured at the bottom of the lesion ( Fig. 4 , Table 3 ).

**Table 3:** Values of von Mises stress in restorative materials and adhesive system under the axial and oblique load

Restorative materials		Axial load (MPa)	Oblique load (MPa)
1.	Nanohybrid composite (Tetric EvoCeram, Ivoclar Vivadent)	13.799	53.76
1.	Adhesive system	6.661	28.742
2.	Hybrid composite (Z 100, 3M Espe)	13.251	52.342
2.	Adhesive system	6.602	28.465
3.	Compomer (Dyract, Dentsply Caulk)	11.907	47.649
3.	Adhesive system	6.475	27.454
4.	Glass ionomer cement (Ketac Molar Easy Mix, 3M Espe)	11.779	47.364
4.	Adhesive system	6.493	27.571
5.	Mycrohybrid composite (Herculite XR, Kerr)	11.46	46.00
5.	Adhesive system	6.466	27.057
6.	Flowable composite (Tetric flow, Ivoclar Vivadent)	9.393	37.763
6.	Adhesive system	6.342	24.656

The lowest values of stress in the adhesive system were measured on lesions restored with a flowable composite at an axial load of 6.342 MPa, as well as an oblique one of 42.653 MPa.

**Fig. 5:**
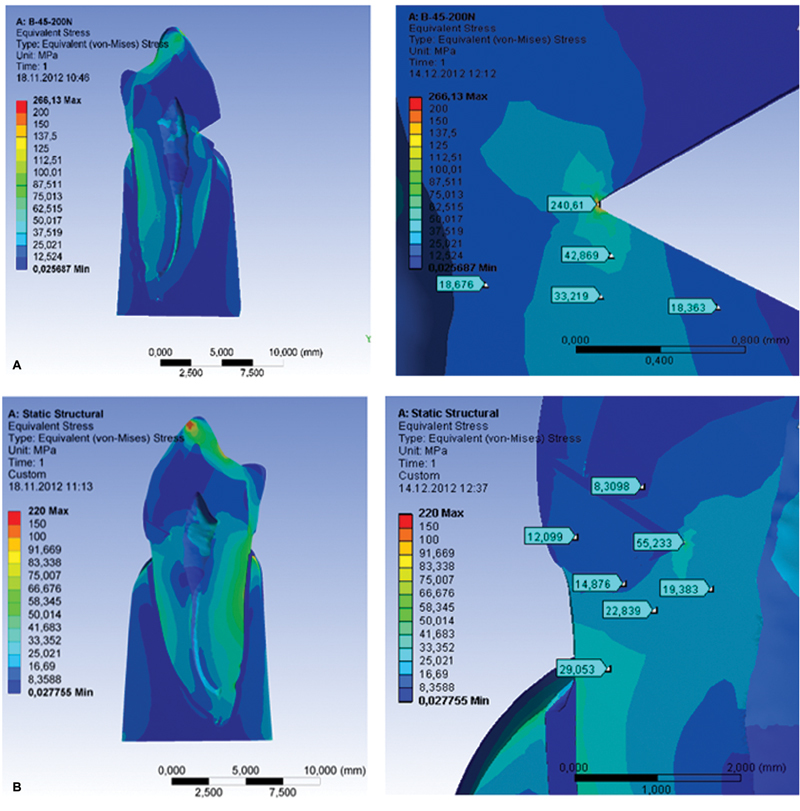
Image of the stress on a sagittal section of a tooth with lesion and restoration under the oblique load of 200 N. ( **A** ) Sagittal section of a tooth with abfraction lesion ( **B** ) Sagittal section of a tooth with restored abfraction lesion.

Restoration of abfraction lesion leads to a significant stress reduction in the apex of lesion, which additionally leads to a redistribution of stress in the tooth. The values of the measured stress of the bottom of abfraction lesion without any restoration are ≈ 240 MPa, while measuring at the same place after the restoration are ≈ 55 MPa, which represents a drastic stress reduction ( Fig. 5 ).

**Fig. 6:**
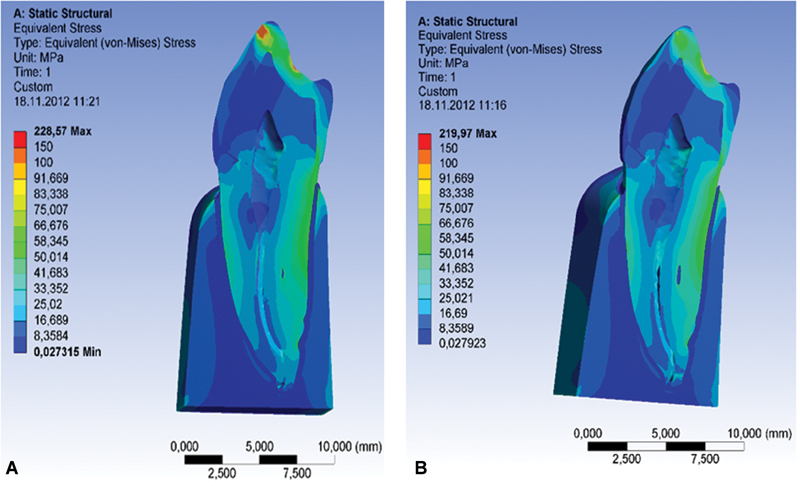
Stress distribution on a sagittal section of a tooth. ( **A** ) Flowable composite (Tetric flow, Ivoclar Vivadent). ( **B** ) Microhybride composite (Herculite XR, Kerr).

The greatest stress in the surrounding dental tissues was measured in the tooth restored with Tetric flow, Ivoclar Vivadent to 228.57 MPa, while the lowest stress was measured in the dental tissue restored with Herculite XR, Kerr 219.97 MPa ( Fig. 6 ).

**Fig. 7:**
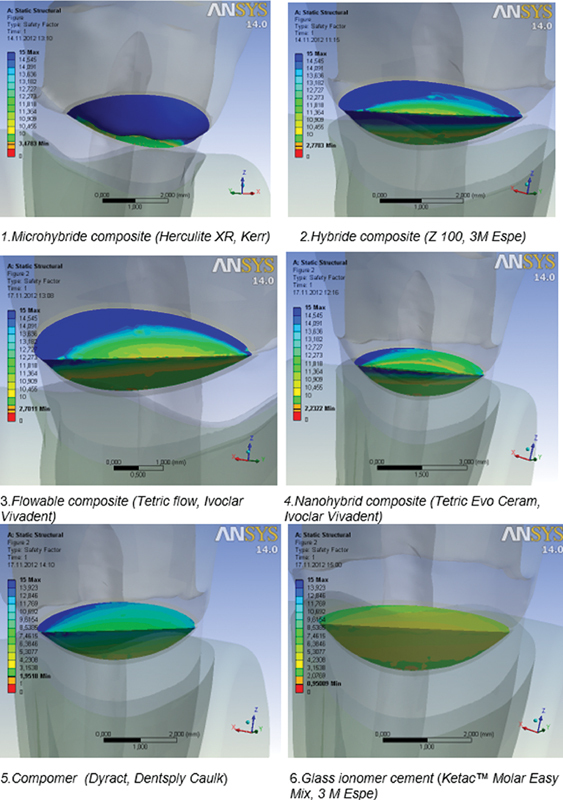
Safety factor values for the restorative materials.

The values of safety factor were determined for the tested restorative materials under the oblique loads of 200 N. The highest values of safety factor of 3.478 MPa were measured in microhybrid composite (Herculite XR, Kerr). Flowable composite (Tetric flow, Ivoclar Vivadent) is on the third place according to the values of safety factor. The values of the safety factor of GIC (Ketac Molar Easy Mix, 3M Espe) are less than 1, which means that the material cannot endure the tested load and it would fracture under the tested pressure ( Fig. 7 , Table 4 ).

**Table 4:** Safety factor values for the tested materials under the oblique load of 200 N

Material		Safety factor
1.	Microhybrid composite (Herculite XR, Kerr)	3.478
2.	Hybrid composite (Z 100, 3M Espe)	2.770
3.	Flowable composite (Tetric flow, Ivoclar Vivadent)	2.701
4.	Nanohybrid composite (Tetric EvoCeram, Ivoclar Vivadent)	2.232
5.	Compomer (Dyract, Dentsply Caulk)	1.951
6.	Glass ionomer cement (Ketac Molar Easy Mix, 3M Espe)	0.950

## Discussion

A good choice of restorative materials is one of most important factors that indicate success in restorative dentistry. Although there are many studies that have analyzed the restorative protocols for NCCL, 11 data on the effect of different materials and restorative techniques are still not clear. Hansen 30 reported that the failure rate of cervical restorations over a 2-year period has been 20%, which is considered as an unacceptable high percentage. Sclerotic dentin is a typical finding on cervical lesions and is consider one of the main reasons of poor adhesion. 10 31

Six materials were analyzed in this study: *Z 100 (3M Espe)* , *Herculite XR (Kerr)* , *Tetric Evo Ceram (Ivoclar Vivadent)* , *Tetric flow (Ivoclar Vivadent)* , *Dyract (Dentsply Caulk)* , and *Ketac Molar Easy Mix (3M Espe)* .

The obtained results show that the distribution of the stress in materials significantly differs depending on the type of load. Oblique loads caused ≈ four times higher stress in restorative materials than the axial ones. The highest stress under both types of loads was measured in the nanohybrid composite (Tetric EvoCeram, Ivoclar Vivadent), while the lowest one was measured in the flowable composite (Tertic flow, Ivoclar Vivadent). The values of the stress measured in all the materials are presented in Fig. 4 and Table 3 .

However, it is interesting that the greatest stress in the surrounding dental tissues was measured in the tooth restored with Tetric flow, Ivoclar Vivadent (material with the lowest stress value), while the lowest stress was measured in the dental tissues restored with Herculite XR, Kerr ( Fig. 5 ). It is obvious that dental tissues suffer greater stresses if the restorative material is of the lower modulus of elasticity.

The analysis also included the adhesive layer of 0.1 mm. Even the minimum discontinuity of the bond in the adhesive layer contributes to greater stress. 32 The stress values in the adhesive layer depend on the type of the material the cervical lesion was restored with. Stresses measured under the axial load are ∼four times lower than those under the oblique load. The highest values were measured under the oblique load in Tetric Evo Ceram, Ivoclar Vivadent, while the lowest ones were measured for Tetric flow, Ivoclar Vivadent ( Table 3 ).

The highest concentration of stress in the restoration and adhesive system of all six materials was observed in the lower (gingival) margin of the filling, while the stress was significantly lower on the upper (occlusal) margin. The values of the stress in the material increased approaching the apex of the lesion. This corresponds to results obtained *in vitro* studies, where the higher shear stresses, microleakage, and fractures were observed at the gingival margin of the restoration. 33 34 The results obtained in clinical studies also refer to a more frequent finding of defects on the lower margin of the restorations. 34 35

The restoration of the lesion leads to a significant reduction of stress on the apex of the lesion. The values of the measured stress of the bottom of noncarious lesion without any restoration are ≈ 240 MPa, while the measuring on the same place after the restoration are ≈ 55 MPa, which represents a drastic reduction of stress ( Fig. 5 ). Results of this study show that the restoration of NCCLs significantly reduce extremely high stress values on their bottom, so the timely treatment can prevent further loss of dental tissues. However, the fact is that simple restorations of cervical lesions do not cure the etiological factor. 11

To find out which of the materials can withstand the stresses they were exposed to, we have to take into consideration one more parameter—the tensile strength of the material. The safety factor enables the calculations of the ratio between the strength and the stress of the material for the purpose of predicting a possible fracture. The safest material in our research was the microhybrid composite (Herculite XR, Kerr) with the highest safety factor of the values of 3.478, then the Hybrid composite (Z 100, 3M Espe), with the values of 2.770 ( Fig. 7 , Table 4 ). This result corresponds to one of a study of Heyman et al, 36 who found after 2-year clinical study that the microfilled composites have tendency to adapt the tooth microflexure and are considered suitable for the restoration of the cervical lesions. Vandewalle and Vigil 37 also recommend microfilled composites in restoration of cervical lesions of the tooth.

In general, long-term clinical studies reported a good clinical performance for most of the resin composites with respect to esthetics, longevity, mechanical properties, surface texture, marginal integrity, anatomic form, and color matching. The loss of the retention of composite fillings in NCCL is probably the combination of more factors such as cervical stress /flexure in the CEJ, settings characteristics and clinical manipulation, tooth location, or the existence of parafunctional habits.

Long-term considering, resin–dentin bond degradation occurs in all the composite systems, and for these purposes, three-step adhesion technique is recommended because it is more reliable compared with two-step self-etch adhesives. 9

Results of this study indicate that the flowable composite (Tetric flow, Ivoclar Vivadent) is the material with the lowest stress values measured under the load, and it is in the third place in terms of safety ( Fig. 7 , Table 4 ). Flowable composites have a low concentration of fillers, low elastic modulus which make them more flexible, but yet, the clinical studies did not find any influence on the differences on modulus of elasticity on retention rate. 9 38 The advantages of flowable composites over Hybrid or Microfilled composites are not clinically proven. Their use could be an advantage in small lesions, where there is no need for sculpturing. 30

Compomer (Dyract, Dentsply Caulk) ( Fig. 7 , Table 4 ) is on the fifth place due to the safety values. Compomers combine the benefits of composites and GICs. The increased elasticity of compomer materials related to GIC promises better performances in stress-exposed cervical part of the tooth. 9 Clinical studies show similar retention rates between compomers and composites, but other parameters such as marginal integrity, color, and surface texture were found to be inferior to those of composites. 9 39

GICs are believed to be a good choice for the restoration of NCCL because they are adhesive, of acceptable biocompatibilities and aesthetics, and they reduce the dentin hypersensitivity, 40 but their poor strength and hardness are the main disadvantages of their usage for these purposes. 11 Our research shows that the values of the obtained safety factor in GIC (Ketac Molar Easy Mix, 3M Espe) are 0.950 (less than 1), which indicates that the material cannot withstand the tested load ( Fig. 7 , Table 4 ). Also, GIC does not provide the possibility of high polishing, it retains more plaque on its surface, and it is abrasive. 11

In this study, the higher stress was observed in more rigid composites under the effect of occlusal forces, while the stress in the surrounding dental tissues was lower. On the other hand, composites with the low elastic modulus are more flexible, but cause more stress in surrounding tissues, which leads to greater tooth deformation. Quality restoration should allow the restored tooth to respond to the load as a healthy tooth. NCCL treatment is a complex procedure, and restoration failure is due to the synergistic action of insufficient material properties, specific biological environment in the cervical area, multifactorial and difficult to manage etiology causing the initial lesion.

## Conclusion

The obtained stress in restorative materials is higher during the effects of oblique load. The highest stress in the restoration and adhesive layer of all the tested materials was observed at the gingival margin of the restoration. The highest stress under both types of loads was measured in nanohybrid composite (Tetric EvoCeram, Ivoclar Vivadent). The lowest stress values were measured in the flowable composite (Tetric flow, Ivoclar Vivadent). Microhybrid composite (Herculite XR, Kerr) with the highest safety factor represents material with the best ratio between the strength and the stress it is exposed. Restoration of NCCLs significantly reduces extremely high values of stress at their apex. Timely treatment can prevent further loss of dental tissue. In the treatment of cervical lesions, it is crucial to determine and eliminate the etiological factor.
